# The *in vitro* effects of Voghera sweet pepper on thyroid cancer cells: modulation of oxidative stress and pro-tumorigenic genes

**DOI:** 10.3389/fnut.2025.1574180

**Published:** 2025-08-25

**Authors:** Francesca Coperchini, Fabrizio De Luca, Alessia Greco, Laura Croce, Elena Franchi, Marsida Teliti, Patrizia Pignatti, Flavia Magri, Maria Grazia Bottone, Mario Rotondi

**Affiliations:** ^1^Department of Internal Medicine and Therapeutics, University of Pavia, Pavia, Italy; ^2^Department of Biology and Biotechnology “L. Spallanzani”, University of Pavia, Pavia, Italy; ^3^Unit of Endocrinology and Metabolism, Laboratory for Endocrine Disruptors, Istituti Clinici Scientifici Maugeri IRCCS, Pavia, Italy; ^4^Allergy and Immunology Unit, ICS Maugeri IRCCS, Pavia, Italy

**Keywords:** Voghera pepper, thyroid cancer, oxidative stress, phytochemical, ROS, anti-oxidant genes

## Abstract

**Background:**

Voghera pepper (VP) extracts were demonstrated to have anti-oxidant ability in several cell types.

**Purpose:**

This study aimed to assess whether VP-extracts could lower oxidative stress and modulate thyroid cancer (TC) cells behavior *in vitro*.

**Methods:**

Extracts were analyzed using the LC-DAD-MS system. Thyroid cell lines, both normal (NHT) and cancerous (TPC-1 and 8505C) were treated with increasing concentrations of Yellow (YVP) and Green (GVP) VP-extracts over time. Viability and proliferation were assessed in all cell types. Changes in Reactive-oxygen-species (ROS) production by TPC-1 and 8505C were assessed by flow-cytometry. The mRNA expression of anti-oxidant mediators (*NFE2L2, HMOX1, SOD2* and *CAT*), epithelial-to-mesenchymal transition markers (*POU5F1, SNAI1, TWIST1, SNAI2* and *VIM*) and thyroid-differentiation-related genes (*NKX2-1* and *PAX8*) were evaluated by RT-PCR.

**Results:**

Treatment with GVP or YVP reduced the viability of TPC-1 and 8505C but not those of NHT, without effects on cells proliferation. GVP and YVP reduced basal and H_2_O_2_-induced ROS production in TC cells. GVP and YVP up-regulated mRNA levels of several anti-oxidant genes. GVP and YVP reduced mRNA of *POU5F1* in TPC-1 and 8505C. Finally, the mRNA of *PAX-8* was reduced by GVP and YVP extracts in TPC-1 and 8505C, while *NKX2-1* was reduced by both GVP and YVP in TPC-1 and only by GVP exposure in 8505C.

**Conclusion:**

This is the first demonstration of the potential beneficial effects of VP extracts in TC in terms of reduction of oxidative stress, increase of antioxidant markers, and modulation of markers of metastasis and de-differentiation in TC cells.

## 1 Introduction

Phytochemicals are bioactive plant compounds. They are derived from various sources, and to date more than a thousand have been discovered. Significant phytochemicals include carotenoids, polyphenols, isoprenoids, phytosterols, saponins, dietary fibers, and certain polysaccharides. These phytochemicals possess antioxidant activities and exhibit antimicrobial, antidiarrheal, anthelmintic, antiallergic, antispasmodic, and antiviral properties (1, 2).

Bioactive phytochemicals have been extensively studied in the last two decades using *in vitro* and *in vivo* models providing important insights into structure–function effects potentially responsible for disease risk reduction (3). Recently, these compounds have become a focal point in cancer therapy due to their wide range of beneficial properties, including anti-inflammatory, antioxidant, and anticancer effects (4–8). Natural product-derived compounds like paclitaxel, doxorubicin, and vincristine are commonly used in cancer treatment due to their potent anti-tumor properties (5). Oxidative stress may be a contributing factor to various human diseases, as oxidation reactions produce free radicals that cause cell damage, alter DNA, and lead to the accumulation of mutations. These evidences encouraged the exploration of compounds with antioxidant properties in pharmacology. The generation of cellular Reactive Oxygen Species (ROS) is triggered by both internal (endogenous) and external (exogenous) stimuli. ROS are naturally produced as byproducts of normal oxygen metabolism and play crucial roles in homeostasis and cell signaling. In several cell types, they are also implicated in damaging cell structures and affecting mitochondrial function (9).

The thyroid is naturally exposed to high levels of ROS, and there is increasing evidence supporting the significant role of oxidative stress in the initiation, promotion, and progression of thyroid cancer (TC) (10–12). TC, the most common endocrine neoplasm, represents ~1% of malignant cancers (13). In the last decades, global TC incidence has continuously increased. Among TC, poorly-differentiated/undifferentiated tumors, pose a significant challenge due to their limited response to standard therapies.

Oxidative stress is an important risk factor for thyroid tumorigenesis. It involves a complex network of interconnected components that can lead to more severe tumor presentation and progression, particularly when associated with specific genetic mutations. Additionally, TC is characterized by an unbalanced antioxidant system, which can promote disease progression. Given the dual role of oxidative stress in both promoting tumor initiation and mediating therapeutic cytotoxicity (14) the antioxidant potential of widely consumed dietary compounds in thyroid cells may provide insights relevant to both prevention and early tumor modulation. Such efforts could help improve clinical outcomes in patients with TC. The potential anticancer effects of phytochemicals on TC are currently the focus of various scientific studies (15).

Recent *in vitro* studies demonstrated anti-oxidant properties of the Voghera pepper (edible part of *Capsicum annuum* L. var. Peperone di Voghera (VP)), a native Lombardy variety of vegetable cultured in Italy. Green and yellow sweet Voghera peppers are frequently consumed and differ in their phytochemical composition. Ripening stages affect the levels of various bioactive compounds, some of which may contribute to antioxidant and anticancer effects (16). This variety of pepper is rich in both vitamin C and carotenoids, has a high nutritional value, and its role in protecting against oxidative damage has been previously reported in different types of cells (17, 18). Based on previous data regarding the antioxidant properties of VP and the well-documented pro-tumorigenic effect of excessive oxidative stress in the development of TC, we aimed to investigate whether VP extracts could reduce oxidative stress and, in turn, modulate TC cells behavior *in vitro*. In addition, the effect of Voghera pepper on genes involved in TC development and progression was also evaluated.

## 2 Materials and methods

### 2.1 Extraction procedure and characterization

Green and yellow sweet peppers were selected as models of early and mid-stage ripening (provided by the Istituto Tecnico Agrario “C. Gallini of Voghera” (PV) and the PepeVo association). The extraction procedure was performed as previously reported (17, 19, 20). Briefly, 15 g of pepper were left in 100 g mixture 1,2-propanediol/water (55 and 45%, respectively) for 2 h at 40 °C. Subsequently, samples were homogenized for 15 min and left to macerate at 40 °C for 2 h. The sample was left to rest for 24 h to allow the solid part to settle, and the supernatant, containing the full range of phenolic acid derivatives and flavonoids extracted from the grams of pepper used, was collected. Subsequently, the extracts were filtered using 0.22 μm filters to ensure an adequate level of sterility for the subsequent analyses, obtaining an extract with a concentration of Voghera pepper or pepper waste equivalent to 0.15 g/mL in a 1,2-propanediol/water mixture. For the characterization of phenolic acid derivatives and flavonoids, extract samples were diluted 1:1 in Dimethyl Sulphoxide (DMSO) and placed in a vial for chromatographic analysis, each measurement was performed in triplicate to ensure the reliability of the results. The solutions were then analyzed using the LC-DAD-MS system composed by an Agilent 1,260 chromatograph equipped with autosampler, diode array series 1,260. At the end of the chromatographic column a “t” junction was used to split the flow equally to Diode Array and Mass spectrometric detector. A Varian MS 500 Ion Trap mass spectrometer was used operating with an Electrospray Ion source collecting data in negative and positive ion mode. The nebulizer gas was Air in negative ion mode and Nitrogen in positive ion mode; the drying gas was nitrogen. Pressure of drying gas was set at 25 psi and nebulizer at 30 psi. Needle was set to 4,500 V, and capillary was set to 80 V. Rf loading was 85% and the data were obtained using the turbo data depending on scan of the instrument that allows the generation of fragmentation tree of ionic species that reach a threshold current value. For the chromatographic separation, an Eclipse XDB C-18 4.6×250 mm 5 μm column was used as the stationary phase. Using a mobile phase composed of a mixture of water with 1% formic acid (A), acetonitrile (B), and methanol (C), the following elution gradient was employed: 0–0.5 min 95:5:0 (A:B:C); 5 min 85:15:0 (A:B:C); 15 min 60:30:10 (A:B:C); 20 min 20:70:10 (A:B:C); 25 min 0:90:10 (A:B:C); 43 min 0:90:10 (A:B:C); 44 min 95:5:0 (A:B:C) flow rate was 400 μL/min. The identification of the detected compounds was carried out based on literature data and fragmentation patterns. UV spectra were acquired at wavelengths of 330, 350, and 254 nm. Rutin and chlorogenic acid were used as reference standards for the general quantification of flavonoids and phenolic acids, respectively. Solutions of reference compounds were prepared in four concentrations (100 μg/mL, 50 μg/mL, 20 μg/mL, 2 μg/mL) and calibration curves were obtained at 350 nm for rutin and 330 nm for chlorogenic acid. Peaks were assigned to flavonoid or chlorogenic acid derivatives on the basis of the UV spectrum and MS data. The data were expressed in μg/mL and reported in the table as mean ± SD of the three replicates.

### 2.2 Primary cultures of normal human thyroid cells and TC cell lines

Primary cultures were established from surgical specimens of human thyroids obtained from five patients who underwent thyroidectomy. The study received approval from the Institutional Board of ICS-Maugeri in Pavia, Italy. Informed consent was signed by all patients. All experiments were conducted in accordance with relevant guidelines and regulations, adhering to the principles of the Declaration of Helsinki. The surgical specimens were chopped into small pieces and incubated with 5 mg/mL of collagenase type I (Sigma-Aldrich; C0130) in Coon’s F12 medium for 4 h at 37 °C. After incubation, the cells were filtered with a cell strainer (70 μm Nylon; Falcon), centrifuged at 1000 rpm for 10 min, washed with 10 mL of Coon’s F12 medium (Sigma-Aldrich), re-centrifuged and then re-suspended in Coon’s 6H medium. Coon’s 6H medium is composed of Coon’s F12 medium (Sigma-Aldrich) with 5% newborn calf serum and six hormones: insulin (5 μg/mL), hydrocortisone (50 μg/mL), transferrin (5 μg/mL), somatostatin (10 ng/mL), gly-his-lysine (10 ng/mL), and bovine TSH (1 mU/mL). The human TC cell lines used in this study are 8505C (from de-differentiated anaplastic thyroid cells) harboring a BRAF (V600E) mutation and TPC-1 (from differentiated papillary thyroid carcinoma) harboring RET/PTC rearrangement. Dulbecco’s Modified Eagle Medium (DMEM) (Sigma, Saint Louis, MO, United States) was supplemented with 10% fetal bovine serum (FBS) (Sigma, Saint Louis, MO, United States), 2 mM L-glutamine and 100 U/mL penicillin/streptomycin (Sigma, Saint Louis, MO, United States) and used for TPC-1, and Roswell Park Memorial Institute (RPMI) 1,640 (Sigma-Aldrich; R0883) supplemented with 10% FBS, 2 mM L-glutamine and 100 U/mL penicillin/streptomycin was used for 8505C.

### 2.3 Viability assay

NHT, TPC-1 and 8505C cells were seeded at 2 × 10^4^ cell/well density in flat 96-well plates (Thermo-Fisher). After adherence (5 h), cells were treated with increasing concentrations of YVP and GVP extracts (0; 0.5; 1; 1.5; 2 mg/mL) for 24, 48 and 72 h, respectively. These concentration ranges were selected based on previous studies conducted on other cell types (17). At the end of each time point, cells were incubated with water-soluble tetrazolium salt (WST-1, Roche). After 30 min the absorbance was measured with a microplate reader (450 nm, Victor NIVO Multimode Plate Reader, PerkinElmer). We measured the absorbance of control (untreated) and treated samples. After measuring optical density (OD), values of treated samples were normalized to the mean OD of the untreated controls (0 mg/mL), which was set at 100%. Results were expressed as percentage of viability relative to control. This normalization resulted in control values without SD, as they were fixed post-calculation. Statistical analysis was performed on these normalized values using one-way ANOVA with *Post Hoc* Bonferroni correction. This normalization approach setting control values at 100% and expressing treated samples as percentages is widely used in cell-based assays, particularly for viability and functional studies, as supported by several methodological references (21, 22). All measurements were performed in at least three independent experiments, each in technical triplicate.

### 2.4 Cell proliferation assay

NHT, TPC-1 and 8505C cells were seeded in a 96-well plate at a density of 3,000 cells per well and incubated with increasing concentrations of YVP and GVP extracts (0; 0.5; 1; 1.5; 2 mg/mL) for 24, 48, 72 h. 200 μL of cold methanol 20% was added to each well to fix cells. Plates were incubated for 20 min at room temperature, then the cells were stained with 0.5% crystal violet dye (C0775; Sigma-Aldrich) for 5 min at room temperature. Cells were then observed under an inverted microscope Olympus BX51 (Olympus, Deutschland GmbH, Hamburg, Germany). 200 μL of 1% sodium dodecyl sulphate (SDS) (436143; Sigma-Aldrich), an anionic detergent, was added to each well to induce the release of crystal violet dye and quantify its optical density (OD). The OD was measured at 570 nm by a microplate reader (450 nm, Victor NIVO Multimode Plate Reader, PerkinElmer). After measuring optical density (OD), values of treated samples were normalized to the mean OD of the untreated controls (0 mg/mL), which was set at 100% using the same approach of viability assay. All measurements were performed in at least three independent experiments, each in technical triplicate.

### 2.5 Evaluation of the production of reactive oxygen species in TPC-1 and 8505C cells

The experimental conditions were designed to evaluate two distinct scenarios: (i) a non-stressed condition, in which cells were exposed to the extracts alone to assess their effect on basal reactive oxygen species (ROS) levels; and (ii) a stressed condition, in which cells were co-treated with H_2_O_2_ and the extracts to evaluate their potential protective role under induced oxidative stress.

- Non-stressed scenario: thyroid cells were treated with GVP and YVP extracts at 1.5 mg/mL for 24 h, the highest non-toxic concentration for all cell types. Separately, as positive control, for evaluating changes in ROS production, cells were treated with 300 μM H_2_O_2_ alone. The oxidative stress assays were carried out under standardized and controlled conditions, including brief time intervals between mixing and application to cells. To investigate the production of ROS by TPC-1 and 8505C, the cell-permeant 2′,7′-dichlorodihydrofluorescein diacetate (H_2_DCFDA) (Sigma Aldrich) was used. Therefore, H_2_DCFDA (1.25 μM) was added for 15 min, and incubated under conditions of CO_2_ 5% and 37 °C. Cells were detached with the only Ethylenediaminetetraacetic acid (EDTA) 0.05%, centrifuged and resuspended with 300 μL in PBS with 0.5% Bovine serum albumin (BSA) (acquisition buffer). BSA was added to the acquisition buffer as it enhances the quality and reliability of fluorescence measurements by preventing nonspecific binding and improving the specificity of fluorescence detection. It also stabilizes cells and contributes to their viability. Additionally, its ability to reduce cell clumping ensures cell-based analysis is accurate and precise (23). We measured fluorescence intensity throughout Fluorescence-activated cell sorting (FACS). The ROS production was calculated as a percentage of the mean Fluorescence Intensity (MFI). The quantification of ROS levels was achieved through the measurement of the MFI of the ROS-sensitive probe, H_2_DCFDA. MFI values were normalized to those of untreated control cells (0 mg/mL), which were set at 100%. ROS levels in extract-treated cells were then expressed as a percentage relative to this baseline, allowing assessment of the extracts’ effects under non-stressed conditions. We assume the untreated control cells as 100% to better represent and visualize the variations in MFI.
- Stressed scenario: in order to evaluate the effect of extracts on H_2_O_2_-induced ROS production (stressed condition), cells were treated for 24 h with GVP or YVP and after this time were treated with H_2_O_2_ in combination with 1.5 mg/mL YVP and GVP for 10 min. The combined pre- and co-treatment protocol was designed to mimic continuous dietary exposure and evaluate antioxidant effects under early tumor-promoting conditions. To investigate ROS changes, FACS analysis using H_2_DCFDA was performed under the same conditions reported previously. ROS production was calculated as a percentage of the mean Fluorescence Intensity (MFI). MFI values were normalized to those of cells treated with H_2_O_2_ only (H_2_O_2_), which were set at 100%. ROS levels in extract-treated cells were then expressed as a percentage relative to this baseline, allowing assessment of the extracts’ effects under stressed conditions. We assume control (H_2_O_2_) as 100% to better represent and visualize the variations in MFI. All measurements were performed in at least three independent experiments, each in technical triplicate.

### 2.6 Real-time polymerase-chain-reaction of the mRNA levels of anti-oxidant, epithelial-to-mesenchimal-transition and thyroid-related genes

RNA was obtained from TPC-1 and 8505C incubated to YVP and GVP 1.5 mg/mL for 24 h, the highest non-toxic concentration for all cell types. Total RNA purification kit (Norgen Biotek, Canada) was used to obtain RNA from thyroid cells; cDNA was then reverse transcribed by a SensiFast cDNA synthesis kit (Bioline, London, United Kingdom). Real-time PCR was performed using Sensi-Fast SYBR Green Hi-ROX kit (Bioline, London, United Kingdom) on StepOne Plus Applied Biosystems real-time PCR system. Primers were obtained from Biomers.net GMBH (Soflinger, Germany). We measured the expression of mRNA levels of anti-oxidant genes (*NFE2L2, HMOX1, SOD2* and *CAT*) in NHT, TPC-1 and 8505C after treatment with GVP or YVP. We measured the expression of mRNA levels in epithelial-to-mesenchimal-transition (EMT)-related genes (*POU5F1, SNAI1, TWIST1, SNAI2,* and *VIM*). mRNA levels were evaluated in TPC-1 and 8505C after treatment with GVP or YVP (1.5 mg/mL). We measured the expression of mRNA levels of thyroid-related gene (*NKX 2–1* and *PAX8*) that were evaluated in TPC-1 and 8505C. *GAPDH* was selected as the reference gene. Gene expression levels were quantified using the comparative Ct (ΔΔCt) method. Ct values of target genes were first normalized to the housekeeping gene *GAPDH* (ΔCt), and then compared to the ΔCt of control samples to obtain relative expression levels (ΔΔCt). Final results were expressed as fold change (2^−ΔΔCt^) relative to untreated control cells set as 1, as they serve as the reference condition for comparison. All measurements were performed in at least three independent experiments, each in technical triplicate.

### 2.7 Statistical analysis

SPSS software was employed (SPSS, Inc., Evanston, IL). Values are reported as mean ± SD unless otherwise noted. One-way ANOVA for normally distributed variables was used for comparing mean group values. *Post-hoc* analysis for multiple comparisons were performed by Bonferroni’s correction. Between-group comparisons were performed by means of Wilcoxon test and Student t-test. The statistical significance was considered for *p* values < 0.05.

## 3 Results

### 3.1 Chemical characterization

Chemical profiles of both GVP and YVP extracts were determined by the measurement of phenolic acid and flavonoid derivatives (Table 1).

**Table 1:** Polyphenols detected (μg/mL) in both GVP and YVP extracts.

Compounds	λ (nm)	MS/MS fragments	Green Voghera pepper (μg/mL)	Yellow Voghera pepper (μg/mL)
Caffeoyl hexoside	210, 240, 295, 325	179, 135	46.99 ± 2.66	16.77 ± 0.45
Quinic acid	220, 295, 325	127, 173, 85	81.64 ± 2.33	21.44 ± 1.04
Feruloyl hexoside	225, 295, 325	193	8.54 ± 0.25	71.21 ± 1.48
4-hydroxycinnamic acid/p-Coumaric acid	210, 295	145	4.44 ± 0.28	9.20 ± 0.37
Total phenolic acid derivatives (μg/mL)			141.60 ± 3.56	118.62 ± 1.90
Luteolin *di-C-*hexoside	265, 356	528, 489	1.30 ± 0.04	2.10 ± 0.05
Quercetin *O-*rhamnoside (7) *O-*glucoside (3)	258, 380	301	1.06 ± 0.03	21.30 ± 0.00
Vicenin-2	220, 330	503, 413, 473, 353	1.77 ± 0.02	2.19 ± 0.06
Apigenin *C*-pentosyl *C-*hexoside	220, 330	357, 164, 133, 96	3.95 ± 0.06	4.89 ± 0.07
Luteolin pentosyl di-hexoside	265, 356	285, 151, 133	18.69 ± 0.07	22.76 ± 0.11
Luteolin-apiosyl hexoside (rut)	265, 356	447, 285	21.11 ± 0.02	18.36 ± 0.01
Luteolin/Apiosyl acetyl-*D*-hexoside	265, 356	579, 489, 459	3.05 ± 0.02	1.66 ± 0.02
*Quercetin 3-O-rhamnoside*	258, 380	301, 151	37.57 ± 0.68	47.87 ± 0.22
Total flavonoid derivatives (μg/mL)			88.49 ± 0.69	122.17 ± 0.27

Rut, rutoside.

In both extracts, phenolic acids and flavonoids were detected. The concentration of total phenolic acid derivatives was higher in the GVP (approximately 119%) extract compared to YVP, whereas total flavonoid derivatives were more abundant in the YVP (approximately 138%) extract than in GVP.

### 3.2 Effect of Voghera pepper extracts on thyroid cells viability

**Figure 1:**
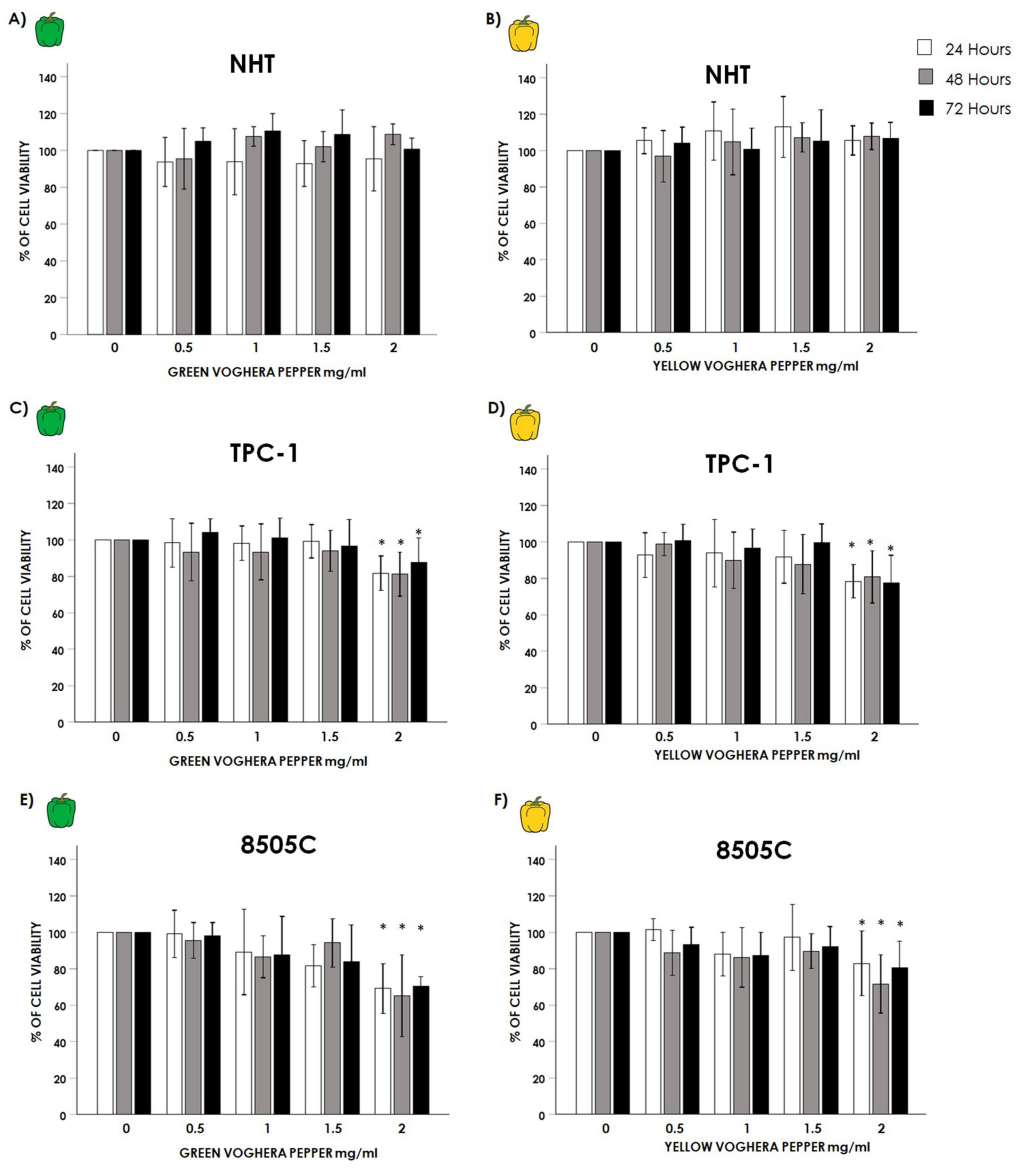
Effect of Voghera pepper extracts on thyroid cell viability. Results of WST-1 employed to assess changes in viability on NHT, TPC-1 and 8505C cells. Bar graphs: y-axis indicates the % of cell viability; x axis indicate the concentrations of Voghera peppers’extracts (mg/mL). Bars show the effect of Green Voghera pepper (GVP) or Yellow Voghera pepper (YVP) after 24 (white bars), 48 (gray bars) and 72 h (black bars). Panels **(A,B)** show the % of cell viability after treating NHT cells with increasing concentration of GVP **(A)** or YVP (**B)**. Panels **(C,D)** show the % of cell viability after treating TPC-1 cells with increasing concentration of GVP **(C)** or YVP **(D)**. Panels **(E,F)** show the % of cell viability after treating 8505C cells with increasing concentration of GVP **(E)** or YVP **(F)**. Bars represent the mean ± SD of normalized values. Control (0 mg/mL) values were set to 100% after normalization; as a result, SD is not shown for control bars. Results were expressed as % of cell viability relative to control. Significant changes between treated samples vs. controls were indicated by *.

Normal Human thyroid cells (NHT), and TC cell lines TPC-1 and 8505C, were treated with increasing concentrations (0; 0.5; 1; 1.5; 2 mg/mL) of GVP or YVP in a time course of 24, 48 and 72 h. The treatment of NHT with either GVP (Figure 1A) or YVP (Figure 1B) did not modify cell viability at any time point at all concentrations tested (GVP ANOVAs: 24 h *F* = 0.304 *p* = 0.873 NS; 48 h *F* = 2.468 *p* = 0.67 NS; 72 h *F* = 2.042 *p* = 0.115 NS); (YVP ANOVAs: 24 h *F* = 1.407 *p* = 0.255 NS; 48 h *F* = 1.223 *p* = 0.332 NS; 72 h *F* = 0.579 *p* = 0.701 NS). Moving to TC cell lines results showed some differences from what observed in NHT. Starting from TPC-1, treatment with GVP at the highest concentration of 2 mg/mL reduced cell viability after 24, 48 and 72 h (Figure 1C: GVP ANOVAs: 24 h *F* = 7.479 *p* < 0.05, *post hoc p* < 0.05 2 mg/mL vs. 0 mg/mL; 48 h *F* = 3.426 *p* < 0.05 *post hoc p* < 0.05 2 mg/mL vs. 0 mg/mL; 72 h *F* = 3.9 *p* < 0,05, *post hoc p* < 0.05 2 mg/mL vs. 0 mg/mL). Similarly, the treatment with 2 mg/mL YVP reduced TPC-1 cell viability after 24, 48 and 72 h (Figure 1D: YVP ANOVAs: 24 h *F* = 4.368 *p* < 0.05, *post hoc p* < 0.05 2 mg/mL vs. 0 mg/mL; 48 h *F* = 4.686 *p* < 0.05 *post hoc p* < 0.05 2 mg/mL vs. 0 mg/mL; 72 h *F* = 9.920 *p* < 0.05, *post hoc p* < 0.05 2 mg/mL vs. 0 mg/mL). The treatment of 8505C with Voghera pepper extracts, again only at the highest concentration of 2 mg/mL, produced a reduction of cell viability similar to that observed in TPC-1, by reducing 8505C viability with GVP after 24, 48 and 72 h (Figure 1E: GVP ANOVAs: 24 h *F* = 7.958 *p* < 0.05, *post hoc p* < 0.05 2 mg/mL vs. 0 mg/mL; 48 h *F* = 17.631 *p* < 0.05 *post hoc p* < 0.05 mg/mL vs. 0 mg/mL; 72 h *F* = 7.646 *p* < 0.05, *post hoc p* < 0.05 2 mg/mL vs. 0 mg/mL) as well as with YVP after 24, 48 and 72 h (Figure 1F: YVP ANOVAs: 24 h *F* = 5.324 *p* < 0.05, *post hoc p* < 0.05 2 mg/mL vs. 0 mg/mL; 48 h *F* = 7.326 *p* < 0.05 *post hoc p* < 0.05 2 mg/mL vs. 0 mg/mL; 72 h *F* = 4.420 *p* < 0.05, *post hoc p* < 0.05 2 mg/mL vs. 0 mg/mL).

### 3.3 Effect of Voghera pepper extracts on thyroid cells proliferation

**Figure 2:**
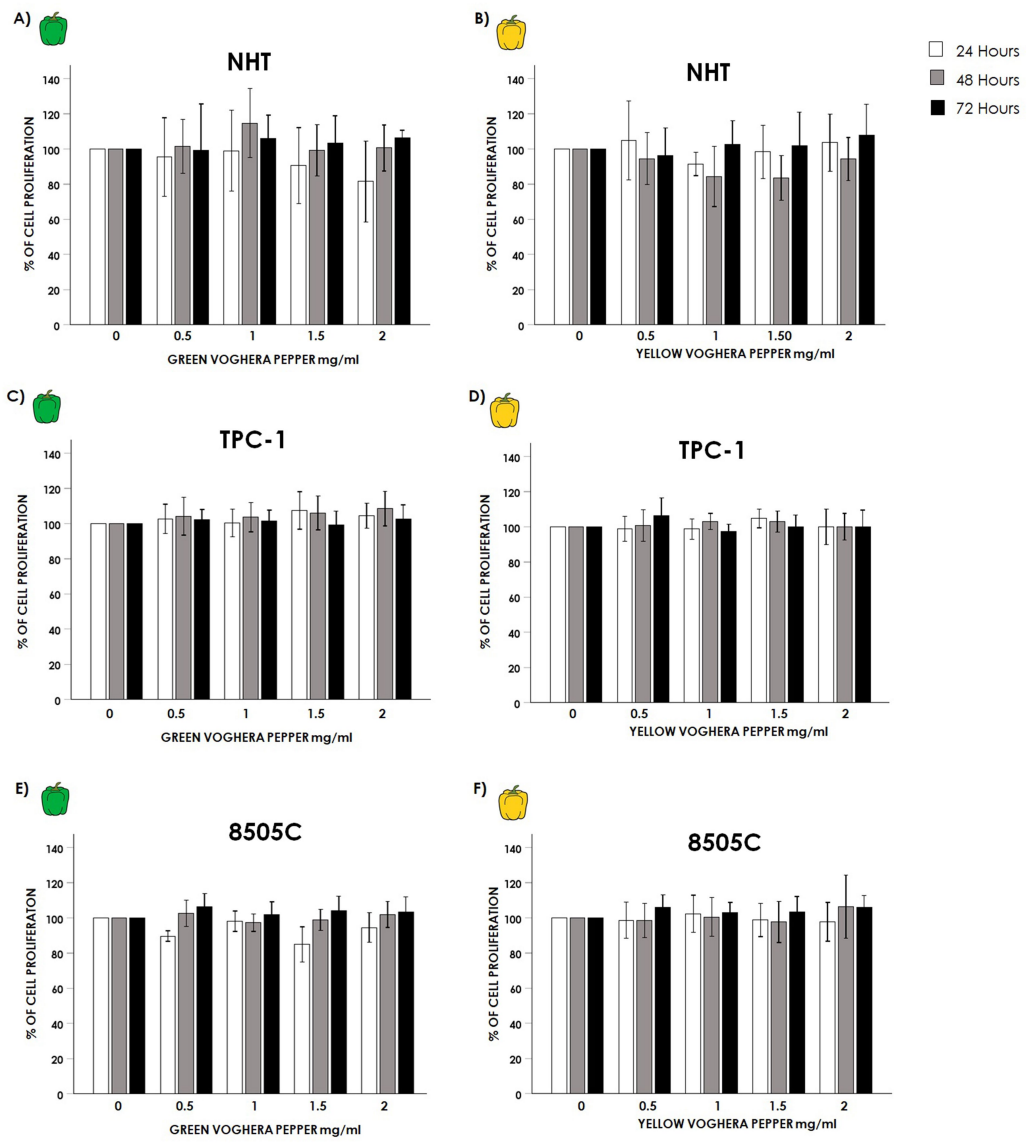
Effect of Voghera pepper extracts on thyroid cells proliferation. Results of crystal violet assay employed to assess changes in the proliferation of thyroid cells. Bar graphs: y-axis indicates the % of cell proliferation; x-axis indicate the concentrations of Voghera peppers (mg/mL). Bars show the effects of Green Voghera pepper (GVP) or Yellow Voghera pepper (YVP) after 24 (white bars), 48 (gray bars) and 72 h (black bars) on cell proliferation. Panels **(A,B)** show the % of cell proliferation after treating NHT cells with increasing concentrations of GVP **(A)** or YVP **(B).** Panels **(C,D)** show the % of cell proliferation after treating TPC-1 cells with increasing concentration of GVP **(C)** or YVP **(D)**. Panels **(E,F)** show the % of cell proliferation after treating 8505C cells with increasing concentration of GVP **(E)** or YVP **(F)**. Bars represent the mean ± SD of normalized values. Control (0 mg/mL) values were set to 100% after normalization; as a result, SD is not shown for control bars. Results were expressed as percentage of cell proliferation relative to control. Significant changes between treated samples vs. controls were indicated by *.

No significant changes in cell proliferation were observed in NHT after treatment with GVP (Figure 2A) (GVP ANOVAs: 24 h *F* = 1.129 *p* = 0.359 NS, 48 h *F* = 1.693 *p* = 0.174 NS, 72 h *F* = 0.369 *p* = 0.829 NS) or YVP (Figure 2B) at any time or concentration tested (YVP ANOVAs: 24 h *F* = 1.067 *p* = 0.387 NS, 48 h F = 1.693 *p* = 0.061 NS, 72 h *F* = 0.623 *p* = 0.649 NS). Similarly, no changes in the proliferation of TC cell lines were found after treatment with VP extracts. Indeed, no changes were observed in the proliferation of TPC-1 cells after treatment with GVP (Figure 2C) (GVP ANOVAs: 24 h *F* = 1.749 *p* = 0.154 NS, 48 h *F* = 1.469 *p* = 0.226 NS, 72 h *F* = 0.611 *p* = 0.656 NS) or with YVP (Figure 2D) (YVP ANOVAs: 24 h *F* = 1.606 *p* = 0.187 NS, 48 h *F* = 0.701 *p* = 0.595 NS, 72 h *F* = 2.420 *p* = 0.061 NS). No changes were found also in 8505C cells after treatment with GVP (GVP ANOVAs: 24 h *F* = 1.735 *p* = 0.157 NS, 48 h *F* = 0.994 *p* = 0.426 NS, 72 h: *F* = 1.692 *p* = 0.167 NS) (Figure 2E) or YVP (YVP ANOVAs: 24 h *F* = 0.396 *p* = 0.810 NS, 48 h *F* = 0.948 *p* = 0.444 NS, 72 h *F* = 1.623 *p* = 0.183 NS) (Figure 2F).

### 3.4 Effect of Voghera pepper extracts on oxidative stress production

The modulation of oxidative stress in TC cells was evaluated by flow cytometry. Two experimental conditions were tested: (i) exposure of cells to extracts alone to assess ROS modulation in non-stressed cells, and (ii) co-treatment with H_2_O_2_ and extracts to evaluate antioxidant effects under induced oxidative stress. The concentration of GVP and YVP extracts of 1.5 mg/mL was chosen since it was the highest non-toxic concentration for all cell types.

**Figure 3:**
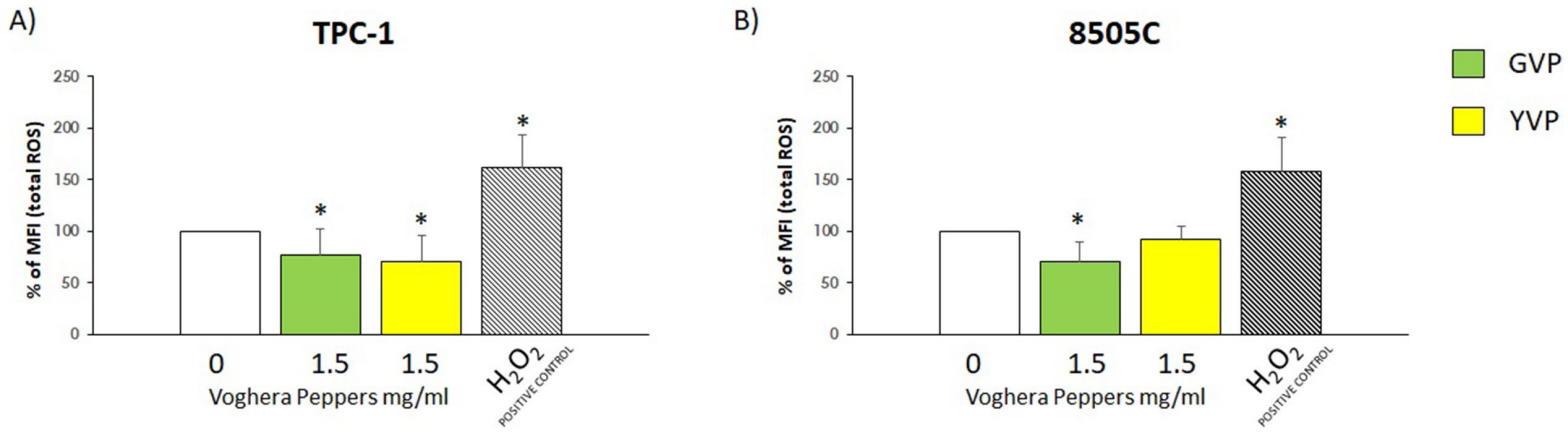
Effect of Voghera pepper extracts on ROS production in non-stressed conditions. Results derived through Fluorescence-activated cell sorting (FACS). Bar graphs: y-axis indicates the % of the mean of fluorescence intensity (MFI) total ROS; x-axis indicates treatment with Voghera peppers (0 or 1.5 mg/mL, for 24 h). Bars show the effect of Green Voghera pepper (GVP) (green bars) and the effect of Yellow Voghera pepper (YVP) (yellow bars) on total ROS production. Panel **(A)** shows the effect of Voghera peppers on total ROS production (in terms of changes of % of control) in TPC-1 after 24 h of treatment with GVP or YVP. Striped bar indicates the treatment with H_2_O_2_ that served as positive control for ROS production by TPC-1. Panel **(B)** shows the effect of Voghera peppers on total ROS production (in terms of changes of % of control) in 8505C after 24 h of treatment with GVP or YVP. Striped bar indicates the treatment with H_2_O_2_ that served as positive control for ROS production by 8505C. Bars shows MFI values that were normalized to those of untreated control cells, set as 100%, and expressed as percentage of ROS relative to control (0). Bars represent the mean ± SD of normalized values. Control values were set to 100% after normalization; as a result, SD is not shown for control bars. Significant changes between treated samples vs. controls were indicated by *.

**Figure 4:**
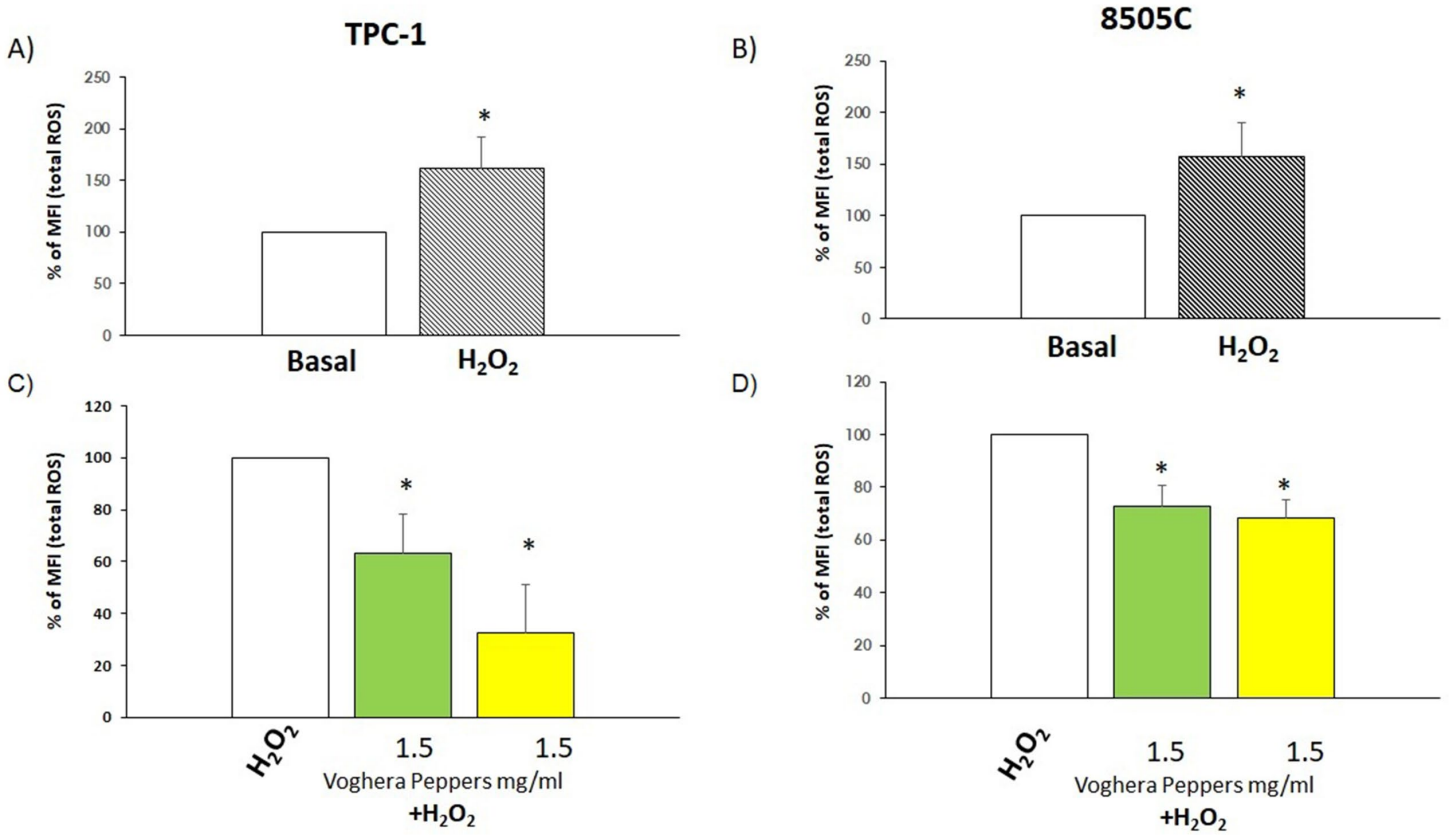
Effect of Voghera pepper extracts on ROS production in stressed conditions (combination with H_2_O_2_). Results derived through Fluorescence-activated cell sorting (FACS). Bar graphs: y-axis indicates the % of the mean of fluorescence intensity (MFI); **(A,B)** X-axis indicates basal ROS production in thyroid cancer cells (white bar) and a ROS increase after exposure to H_2_O_2._ Results are expressed as percentage % calculated on the MFI of untreated samples. **(C,D)** X-axis indicates treatment with GVP (green bar) or YVP (yellow bar) in combination with H_2_O_2_. White bar indicate treatment with H_2_O_2_ alone. Bars shows the percentage of mean of Fluorescence Intensity (MFI). Bars shows MFI values that were normalized to those of H_2_O_2_ treated control cells, set as 100%, and expressed as percentage of ROS relative to H_2_O_2_ treated control. Bars represent the mean ± SD of normalized values. Control values were set to 100% after normalization; as a result, SD is not shown for control bars. Significant changes between treated samples vs. controls were indicated by *.

- Non-stressed scenario: changes in total ROS production were assessed after treatment with Voghera pepper extracts for 24 h. As shown in Figures 3A,B, the analysis of % MFI showed a reduction of ROS production in TPC-1 and 8505C cells treated with (green bar) compared to untreated group (white bar). On the other hand, the treatment with YVP produced a significant reduction of ROS only in TPC-1 (Figure 3A, yellow bar) compared to untreated group (white bar).
- Stressed scenario: in order to evaluate the effect of Voghera pepper extracts on stimulated ROS production (stressed condition), TPC-1 and 8505C cells were pre-exposed for 24 h with 1.5 mg/mL GVP or YVP extracts and then co-exposed with 300 μM H_2_O_2_ in combination with 1.5 mg/mL YVP and GVP for 10 min. This combined pre- and co-exposure protocol was used to reflect *in vitro* a real-life physiological scenario in which, bioactive compounds may be present systemically both prior to and during oxidative insult. As shown in Figures 4A,B), the treatment with H_2_O_2_ increased the basal levels of ROS in both cell types. Interestingly, the co-exposure of GVP or YVP extracts with H_2_O_2_ significantly reduced the H_2_O_2_-induced ROS production in TPC-1 and 8505C cells (Figures 4C,D).

### 3.5 Effect of Voghera pepper extracts on the mRNA levels of anti-oxidant genes

In order to assess the effects of Voghera pepper extracts in terms of anti-oxidant genes expression, the mRNA levels of *NFE2L2*, *HMOX1*, *SOD2* and *CAT* genes were evaluated after treatment with GVP or YVP.

**Figure 5:**
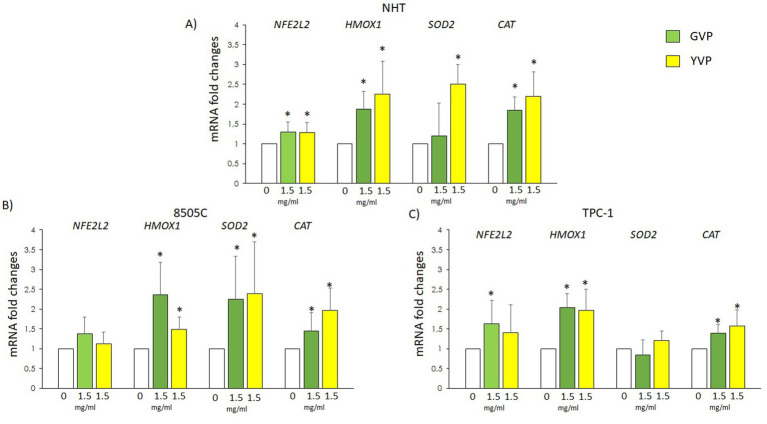
Effect of Voghera pepper extracts on mRNA levels of oxidative stress related genes in thyroid cells. Results of RT-PCR experiments in NHT **(A)**, 850C **(B)** and TPC-1 **(C)** cells treated with Voghera peppers at 0 (white bars) or 1.5 mg/mL for 24 h. Bar graphs: y-axis indicates the fold changes in the mRNA levels of *NFE2L2, HMOX1, SOD2* and *CAT* normalized for *GAPDH* and analyzed using the ΔΔCt method (further normalized for untreated samples, see Material and methods); x-axis indicate treatments, where Green bars represent treatment with Green Voghera pepper (GVP) and Yellow bars represent treatment with Yellow Green pepper (YVP). Significant changes between treated samples vs. controls were indicated by *.

In NHT, GVP increased the mRNA levels of *NFE2L2*, *HMOX1* and *CAT* (Figure 5A, green bars) while YVP increased, also those of *SOD2* (Figure 5A, yellow bars) as compared with untreated samples (white bars). In 8505C cells, treatment with GVP (green bars) or YVP (yellow bars) induced a significant increase in the mRNA expression levels of *HMOX1*, *SOD2* and *CAT* with no effects on *NFE2L2* mRNA (Figure 5B) as compared with untreated samples (white bars). In TPC-1 cells, treatment with GVP (green bars) induced a significant increase in the mRNA expression levels of *NFE2L2, HMOX1 and CAT* with no effects on *SOD2* mRNA as compared with untreated samples (white bars), while treatment with YVP increased the mRNA expression levels of *HMOX1* and *CAT* without significant effect on *NFE2L2* and *SOD2* (Figure 5C) as compared with untreated samples (white bars).

### 3.6 Effect of Voghera pepper extracts on the expression of genes involved in epithelial-to-mesenchimal-transition

**Figure 6:**
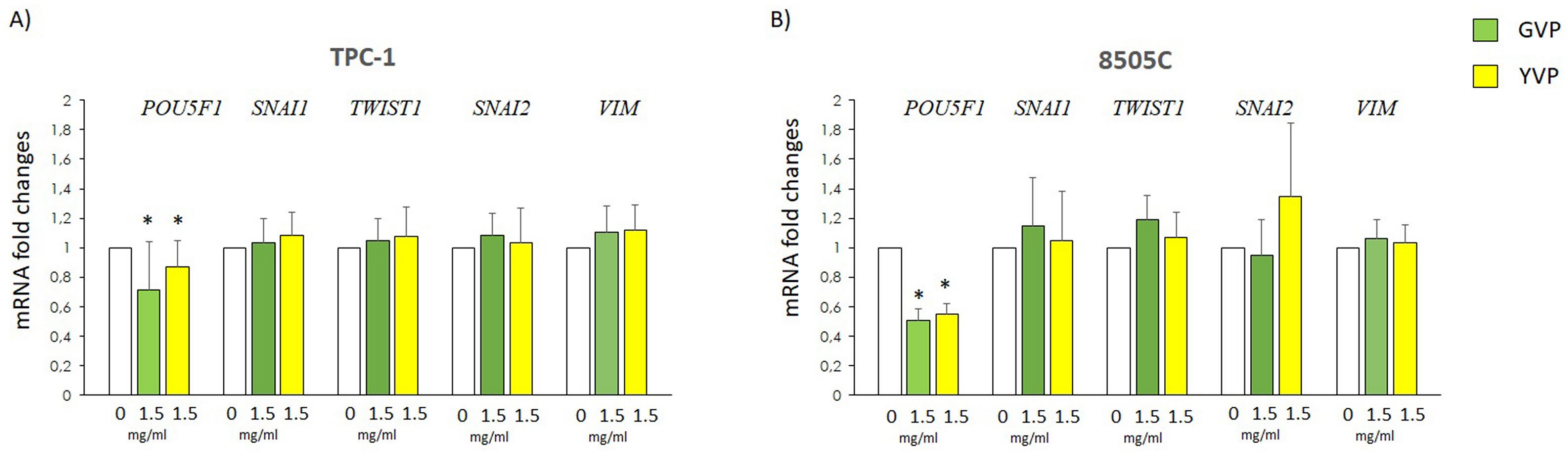
Effect of Voghera pepper extracts on mRNA levels of EMT genes in thyroid cancer cells. Results of RT-PCR experiments in TPC-1 **(A)** and 850C **(B)** cells treated with Voghera peppers at 0 (white bars) or 1.5 mg/mL for 24 h. Bar graphs: y-axis indicates the fold-changes in mRNA levels of *POU5F1*, *SNAI1, TWIST1, SNAI2, VIM* normalized for GAPDH and analyzed using the ΔΔCt method (further normalized for untreated samples, see material and methods). X-axis indicate the treatments. Green bars represent treatment with Green Voghera pepper (GVP); Yellow bars represent treatment with Yellow Green pepper (YVP). Significant changes between treated samples vs. controls were indicated by *.

The potential changes of EMT-related genes mRNA levels were evaluated in TPC-1 and 8505C after treatment with GVP (green bars) or YVP (yellow bars) (Figures 6A,B). The results showed that treatment with GVP or YVP significantly reduced mRNA of *POU5F1* in both cell types, while the mRNA of other EMT-related genes was not modified (Figures 6A,B) as compared with untreated samples (white bars).

### 3.7 Effect of Voghera pepper extracts on the mRNA levels of genes associated with thyroid carcinogenesis

**Figure 7:**
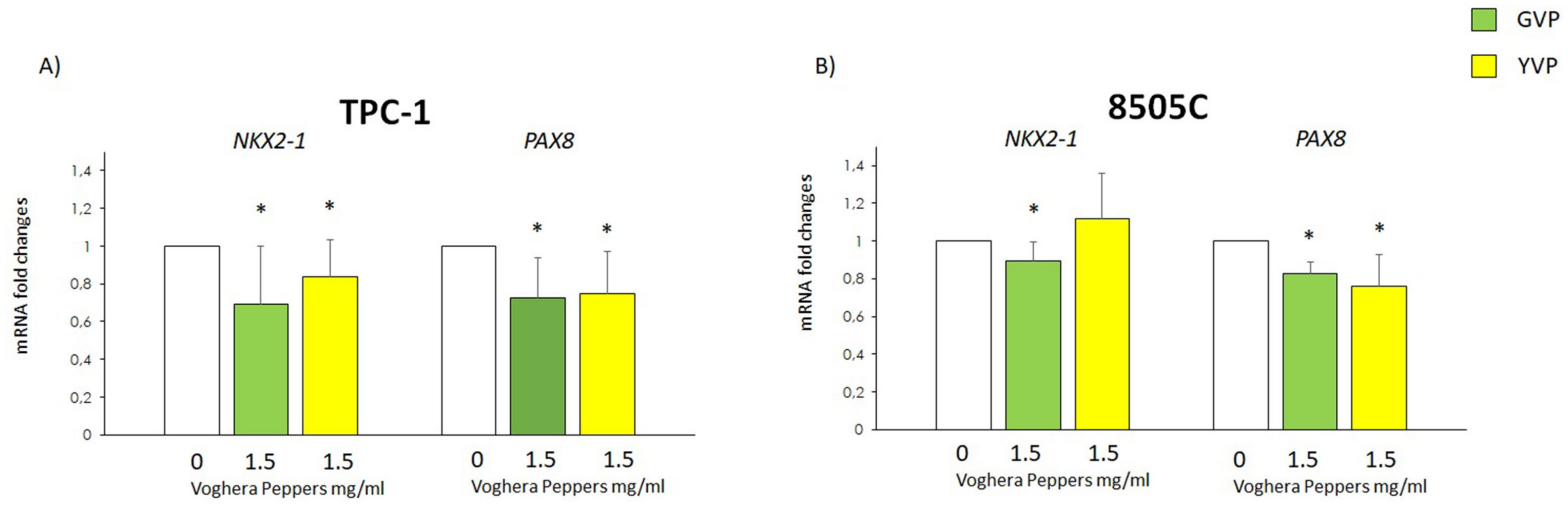
Effect of Voghera pepper extracts on mRNA levels of *NKX2-1* and *PAX8.* Results of RT-PCR experiments in TPC-1 **(A)** and 850C **(B)** cells treated with Voghera peppers at 0 (white bars) or 1.5 mg/mL for 24 h. Bar graphs: y-axis indicates the fold-changes in mRNA levels of *NKX2-1* and *PAX8* normalized for *GAPDH* and analyzed using the ΔΔCt method (further normalized for untreated samples, see materials and methods). The x-axis indicates treatments: Green bars represent treatment to Green Voghera pepper (GVP); Yellow bars represent treatment to Yellow Green pepper (YVP). Bars show results of RT-PCR. Significant changes between treated samples vs. controls were indicated by *.

In order to assess if the treatment with Voghera pepper extract could modify the expression of some genes involved in thyroid carcinogenesis, the potential changes in *NKX2-1* and *PAX8* mRNA levels were evaluated in TPC-1 and 8505C after treatment with GVP (green bars) or YVP (yellow bars) (Figures 7A,B). The results showed that treatment with GVP or YVP, reduced the mRNA of both *NKX2-1* and *PAX8* in TPC-1 as compared with untreated samples (white bars) (Figure 7A). On the other hand, in 8505C, a reduction of the mRNA of both genes was obtained only after treatment with GVP (green bars), while the treatment with YVP (yellow bars) reduced only *NKX2-1* gene mRNA levels as compared with untreated samples (white bars) (Figure 7B).

## 4 Discussion

This is the first study investigating the potential effects of Voghera pepper extracts on thyroid cells *in vitro.* The design of the present study allowed us to evaluate the effects of these substances on crucial aspects of TC development and progression. Indeed, the study focused on the effects of Voghera pepper extracts on cells viability, proliferation, oxidative stress, as well as on changes in the mRNA levels of genes involved in both thyroid tumorigenesis and metastasis.

The results of the viability experiments showed that both GVP and YVP induced a reduction of TC cell lines viability at the highest concentration (Figures 1C–F) while no changes were found in normal thyroid cells (Figures 1A,B). The proliferation of normal and cancer thyroid cells was not modified after treatment with GVP or YVP (Figure 2). These results would suggest a certain cytotoxicity of Voghera pepper extracts on TC cells (even if only at the highest concentration) without adverse effects on normal thyroid cells. The observed reduction in cell viability, despite unchanged proliferation rates, suggests that the extracts may trigger non-proliferative cell stress responses or early apoptotic events, not immediately affecting cell division.

When oxidative stress was evaluated, treatment with GVP or YVP reduced total ROS levels in thyroid cancer cell lines under non-stressed conditions (Figures 3A,B). Additionally, a co-treatment with H_2_O_2_ was used to mimic a physiologically relevant oxidative challenge and to assess the extracts’ potential protective effects. The observed ROS reduction suggests that the extracts may support cellular antioxidant responses (Figures 4C,D). This experimental approach was not designed to distinguish between pre-exposure-driven activation of defense pathways and direct ROS scavenging, but rather to simulate continuous dietary exposure to antioxidant compounds. The primary aim was to evaluate redox modulation during early stages of tumorigenesis, rather than therapeutic efficacy in advanced cancer. However, this approach does not allow us to discriminate whether the observed effects are due to direct H_₂_O_₂_ scavenging or to the activation of intracellular antioxidant pathways. Future studies using separate pre-exposure and co-exposure models will be required to clarify the relative contribution of redox signaling modulation vs. chemical scavenging mechanisms. The reduction in oxidative stress may seem paradoxical in the context of the reduced viability observed in the present study. However, several natural compounds exhibit both antioxidant and cytotoxic effects, often through redox-independent pathways, such as modulation of signaling cascades (e.g., MAPK, NF-κB) or direct interference with cell survival regulators (24–26).

Although a direct comparison of ROS levels between normal and tumor thyroid cells would provide additional insight, this analysis was not included due to the limited yield and replicative capacity of primary human thyroid cells, which hindered reliable quantification under our assay conditions. To mitigate this, we focused on assessing mRNA levels of key antioxidant enzymes (*NFE2L2, HMOX1, SOD2, CAT*) in both thyroid normal and cancer cells to gain insight into their oxidative stress response ability (Figure 5). These data offer an indication of redox-modulatory activity in a physiological context. These enzymes are associated with the degradation of products derived from oxidative stress, protecting cells from DNA damage and apoptosis. Nuclear factor erythroid 2-related factor 2 is responsible for regulation of several antioxidant enzymes. The protein heme oxygenase 1 is a well-established cytoprotective protein that provides its protective effects through the anti-apoptotic and antioxidant properties of heme degradation catabolites (27). Superoxide dismutase 2 performs dismutase activity in the mitochondrial matrix by scavenging O_2_^−^ and effectively eliminates ROS within sites of their generation (28, 29). Catalase is an important antioxidant enzyme that significantly reduces oxidative stress by breaking down hydrogen peroxide into water and oxygen (30, 31). The observed upregulation of *NFE2L2, HMOX1, SOD2* and *CAT* following extract treatment suggests an engagement of antioxidant defense pathways (Figure 5). In addition, the increase in mRNA levels of these genes in NHT would indicate a further potential benefit of Voghera pepper extract in protecting thyrocytes from oxidative stress (Figure 5A). However, the role of these genes in TC remains debated due to the dual role of oxidative stress in cancer development. Indeed, oxidative stress could activate a variety of signaling pathways, which in turn can favor the development of cancer (19). Antioxidants could potentially mitigate early tumorigenic events or limit oxidative stress-driven mutagenesis, which would be beneficial particularly in the context of chemoprevention or in non-tumoral cells. In addition, several studies highlighted that increasing the expression of genes normally involved in anti-oxidant pathways could be beneficial also in TC cells. Just to give a few examples, antioxidant catalase expression was found to be significantly reduced in human thyroid tumors (32) indicating an imbalance of oxidant/antioxidant system in TC (33, 34). Moreover, a low expression of *SOD2* was correlated with a reduction in the survival of patients with aggressive TC. In addition, in mice with aggressive TC, overexpression of *SOD2* reduced tumor proliferation and mortality rates, whereas its deficiency enhanced tumor growth (35).

On the other hand, other studies reported that the overexpression of these genes could be related to a more aggressive behavior of TC. For example, the increased levels of *HMOX1* have been associated to tumor aggressiveness and presence of serine/threonine-protein kinase B-Raf (BRAF)^V600E^ mutation (36, 37). Moreover, in cancers, particularly during treatment with agents that rely on pro-oxidant mechanisms to induce cell death, antioxidants may blunt therapeutic efficacy or even promote tumor cell survival.

Although VP extracts increased the expression of antioxidant enzymes in TC cells, this did not translate into enhanced cell viability or proliferation. On the contrary, cell viability was reduced at higher concentrations. These findings suggest that the antioxidant effects may not confer protection but rather reflect a redox shift potentially unfavorable to tumor cell survival. Moreover, the observed antioxidant effects in TC cells should not be interpreted within a chemotherapeutic framework. Rather, they reflect the potential of sweet pepper extracts to mitigate oxidative imbalance under basal or pro-oxidant conditions, which may be relevant in early disease stages or in preventing tumor-promoting inflammation.

Given the dual role of oxidative stress in cancer, such modulation could influence cell signaling, phenotype, or treatment sensitivity. In this context, mild antioxidant activation may shift tumor cells toward less aggressive behavior. Further studies are needed to clarify these effects, particularly in combination with chemotherapeutic agents.

Lastly, we investigated if Voghera pepper extract could exert some effects on some markers of tumor aggressiveness. At first instance, we analyzed the effect of GVP and YVP on several markers of epithelial-to-mesenchymal transition finding no changes in the mRNA levels on most of the selected genes, excluding *POU5F1* which was reduced by the treatment with both extracts in both TPC-1 and 8505C cells (Figure 6).

*POU5F1* plays a crucial role in the self-renewal and pluripotency of embryonic and germline stem cells, as well as embryonic cancer cells. Additionally, *POU5F1* promotes tumor proliferation, migration, invasion, and tumorigenicity (38). The reduction of the mRNA levels of *POU5F1* in TC cells could be considered a further potential beneficial effect of Voghera pepper extract (Figures 6A,B).

The potential effect of Voghera pepper extracts on TC was evaluated also in terms of thyroid carcinogenesis by assessing changes in the mRNA levels of *PAX8* and *NKX2-1* (Figure 7), two pivotal genes involved in thyroid organogenesis (39). The thyroid primordium organ develops from the embryonic foregut and, at early stage of differentiation, it expresses the transcription factor *NKX2-1.* The expression of this gene signifies the differentiation of primordial endodermal cells toward their final thyroid fate (40). During the organization of cells into characteristic thyroid follicles, which are essential for the biosynthesis and regulation of thyroid hormone secretion, the transcription factor PAX8 plays a crucial role in these processes. The involvement of *PAX8* in early thyroid development has been well-documented (41, 42). The expression of these genes is crucial for the proper differentiation of primordial thyroid cells into thyrocytes, and their overexpression is associated with the aggressiveness of TC. The treatment of TC cells with GVP led to a small but significant reduction of *NKX2-1* and *PAX8* expression in both TC cell lines. On the other hand, treatment with YVP reduced *NKX2-1* in TPC-1 but not 8505C and *PAX8* in both TC cell types (Figures 7A,B). The different responses of TPC-1 and 8505C cells to Voghera pepper extracts likely reflect their distinct levels of differentiation and genetic backgrounds. These factors influence redox regulation and gene expression, explaining the variable modulation of antioxidant, EMT, and thyroid-specific genes observed between the two models. Our findings are in line with previous reports demonstrating anti-tumoral properties of *Capsicum annuum* extracts (another variety of pepper) in various cancer cell lines, including prostate, breast, and colon cancers (43, 44). However, to our knowledge, this is the first study investigating green and yellow sweet pepper extracts in TC models. While some studies have focused on red pepper varieties or isolated compounds such as capsaicin or carotenoids, our work contributes novel insights into the potential modulatory effects of less-studied ripening stages.

Of note, some differences were found when cells were treated with GVP or YVP in our experiments. We selected green and yellow sweet peppers as models of early and mid-stage ripening, respectively, which are frequently consumed and differ in their phytochemical composition. It is well-established that ripening stages affect the levels of various bioactive compounds (including flavonoids, carotenoids, and phenolic acids), some of which may contribute to antioxidant and anticancer effects (16). The differences in the composition of the two extracts is the probable cause of their different effects. This hypothesis is substantiated by the distinct profiles of polyphenolic compounds, i.e., phenolic acid and flavonoid derivatives, quantified in the two analyzed extracts of Voghera pepper. In fact, as supported by recent literature, the high levels of quinic acid measured in GVP may contribute to its anti-inflammatory and antioxidant effects (45), while the elevated levels of quercetin detected in YVP could support the beneficial effects observed for this pepper variety (46).

It should be emphasized that while the extract concentrations used were appropriate for our cellular studies, *in vivo* bioavailability should indeed be evaluated in future research. Such investigations would provide crucial insights into the pharmacokinetics and pharmacodynamics of these extracts, including how they are absorbed, distributed, metabolized, and excreted in the body. Key parameters to assess would include not only bioavailability but also volume of distribution, clearance, and half-life.

The potential benefits derived from the treatment with Voghera pepper extract was demonstrated also in other cells types in terms of anti-oxidative stress properties and also anti-aging effects (17, 19). The present study confirmed the anti-oxidative stress effect of Voghera pepper also on thyroid cells.

## 5 Conclusion

In conclusion, the results of the present *in vitro* study first demonstrated the potential beneficial effects of Voghera pepper extracts for thyroid cells in terms of: (i) induction of cytotoxicity in TC cells without affecting normal thyroid cells, (ii) reduction of oxidative stress with a parallel increase of antioxidant markers, (iii) modulation of markers of metastasis and de-differentiation in TC cells. The induction of antioxidant response genes in thyroid tumor cells by Voghera pepper extracts may reflect a modulation of redox homeostasis rather than a protective effect *per se*. Given the dual role of oxidative stress in cancer, such modulation could influence cell signaling, phenotype, or treatment sensitivity. In this context, mild antioxidant activation may shift tumor cells toward less aggressive behavior. Further studies are needed to clarify these effects, particularly in combination with chemotherapeutic agents. The lack of studies regarding the potential effects of sweet pepper extract on thyroid cells highlights the novelty of our results as well as the relevance of exploring dietary, widely consumed vegetables in thyroid cancer research. In view of the numerous data regarding the potential anti-cancer effect of diverse phytochemicals also in TC, we would encourage further studies to investigate the effects of Voghera pepper extracts in this field.

## Data Availability

The raw data supporting the conclusions of this article will be made available by the authors, without undue reservation.

## References

[1] Sharma BR Kumar V Gat Y Kumar N Parashar A Pinakin DJ. Microbial maceration: a sustainable approach for phytochemical extraction. 3 Biotech. (2018) 8:401. doi: 10.1007/s13205-018-1423-8, PMID: 30221114 PMC6128812

[2] Jaeger R Cuny E. Terpenoids with special pharmacological significance: a review. Nat Prod Commun. (2016) 11:1373–90. doi: 10.1177/1934578X1601100946, PMID: 30807045

[3] Dillard C. Phytochemicals: nutraceuticals and human health. J Sci Food Agric. (2000) 80:1709–811. doi: 10.1002/1097-0010(20000915)80:12<1744::AID-JSFA725>3.0.CO;2-W

[4] Unnikrishnan Meenakshi D Narde GK Ahuja A Al Balushi K Francis AP Khan SA. Therapeutic applications of Nanoformulated resveratrol and quercetin phytochemicals in colorectal Cancer-an updated review. Pharmaceutics. (2024) 16:761. doi: 10.3390/pharmaceutics16060761, PMID: 38931884 PMC11206904

[5] Burgers LD Fürst R. Natural products as drugs and tools for influencing core processes of eukaryotic mRNA translation. Pharmacol Res. (2021) 170:105535. doi: 10.1016/j.phrs.2021.105535, PMID: 34058326

[6] Newman DJ. Natural products and drug discovery. Natl Sci Rev. (2022) 9:206. doi: 10.1093/nsr/nwac206, PMID: 36404871 PMC9668068

[7] Vitale GA Geibel C Minda V Wang M Aron AT Petras D. Connecting metabolome and phenotype: recent advances in functional metabolomics tools for the identification of bioactive natural products. Nat Prod Rep. (2024) 41:885–904. doi: 10.1039/D3NP00050H, PMID: 38351834 PMC11186733

[8] Atanasov AG Zotchev SB Dirsch VM Supuran CT Taskforce INPS. Natural products in drug discovery: advances and opportunities. Nat Rev Drug Discov. (2021) 20:200–16. doi: 10.1038/s41573-020-00114-z, PMID: 33510482 PMC7841765

[9] Devasagayam TP Tilak JC Boloor KK Sane KS Ghaskadbi SS Lele RD. Free radicals and antioxidants in human health: current status and future prospects. J Assoc Physicians India. (2004) 52:794–804. 15909857

[10] Muzza M Pogliaghi G Colombo C Carbone E Cirello V Palazzo S. Oxidative stress correlates with more aggressive features in thyroid Cancer. Cancers. (2022) 14:857. doi: 10.3390/cancers14235857, PMID: 36497339 PMC9737385

[11] Faria CC Fortunato RS. The role of dual oxidases in physiology and cancer. Genet Mol Biol. (2020) 43:e20190096. doi: 10.1590/1678-4685/GMB-2019-0096, PMID: 32453337 PMC7265977

[12] Wang D Feng JF Zeng P Yang YH Luo J Yang YW. Total oxidant/antioxidant status in sera of patients with thyroid cancers. Endocr Relat Cancer. (2011) 18:773–82. doi: 10.1530/ERC-11-0230, PMID: 22002574 PMC3230112

[13] Balajam NZ Mousavian AH Sheidaei A Gohari K Tavangar SM Ghanbari-Motlagh A. The 15-year national trends of endocrine cancers incidence among Iranian men and women; 2005-2020. Sci Rep. (2023) 13:7632. doi: 10.1038/s41598-023-34155-2, PMID: 37164997 PMC10172312

[14] Huang R Chen H Liang J Li Y Yang J Luo C. Dual role of reactive oxygen species and their application in Cancer therapy. J Cancer. (2021) 12:5543–61. doi: 10.7150/jca.54699, PMID: 34405016 PMC8364652

[15] Kaczmarzyk I Nowak-Perlak M Woźniak M. Promising approaches in plant-based therapies for thyroid cancer: an overview of in vitro, in vivo, and clinical trial studies. Int J Mol Sci. (2024) 25:463. doi: 10.3390/ijms25084463, PMID: 38674046 PMC11050626

[16] Zamora R Navarro JL Gallardo E Hidalgo FJ. Chemical conversion of alpha-amino acids into alpha-keto acids by 4,5-epoxy-2-decenal. J Agric Food Chem. (2006) 54:6101–5. doi: 10.1021/jf061239n, PMID: 16881723

[17] Gola F Gaiaschi L Roda E De Luca F Ferulli F Vicini R. Voghera sweet pepper: a potential ally against oxidative stress and aging. Int J Mol Sci. (2023) 24:782. doi: 10.3390/ijms24043782, PMID: 36835192 PMC9959306

[18] Mennella G D'Alessandro A Francese G Fontanella D Parisi M Tripodi P. Occurrence of variable levels of health-promoting fruit compounds in horn-shaped Italian sweet pepper varieties assessed by a comprehensive approach. J Sci Food Agric. (2018) 98:3280–9. doi: 10.1002/jsfa.8831, PMID: 29230827

[19] De Luca F Gola F Azzalin A Casali C Gaiaschi L Milanesi G. A lombard variety of sweet pepper regulating senescence and proliferation: the Voghera pepper. Nutrients. (2024) 16:1681. doi: 10.3390/nu16111681, PMID: 38892614 PMC11174795

[20] Volkov VA Voronkov MV Misin VM Fedorova ES Rodin IA Stavrianidi AN. Aqueous propylene glycol extracts from medicinal plants: chemical composition, antioxidant activity, standardization, and extraction kinetics. Inorg Mater. (2021) 57:1404–12. doi: 10.1134/S0020168521140120

[21] Fotakis G Timbrell JA. In vitro cytotoxicity assays: comparison of LDH, neutral red, MTT and protein assay in hepatoma cell lines following exposure to cadmium chloride. Toxicol Lett. (2006) 160:171–7. doi: 10.1016/j.toxlet.2005.07.001, PMID: 16111842

[22] Perużyńska M Nowak A Birger R Ossowicz-Rupniewska P Konopacki M Rakoczy R. Anticancer properties of bacterial cellulose membrane containing ethanolic extract of Epilobium angustifolium L. Front Bioeng Biotechnol. (2023) 11:1133345. doi: 10.3389/fbioe.2023.1133345 36890919 PMC9986418

[23] Cossarizza A Chang HD Radbruch A Acs A Adam D Adam-Klages S. Guidelines for the use of flow cytometry and cell sorting in immunological studies (second edition). Eur J Immunol. (2019) 49:1457–973. doi: 10.1002/eji.201970107 31633216 PMC7350392

[24] Chauhan A Islam AU Prakash H Singh S. Phytochemicals targeting NF-κB signaling: potential anti-cancer interventions. J Pharm Anal. (2022) 12:394–405. doi: 10.1016/j.jpha.2021.07.002, PMID: 35811622 PMC9257438

[25] Radzka J Łapińska Z Szwedowicz U Gajewska-Naryniecka A Gizak A Kulbacka J. Alternations of NF-κB signaling by natural compounds in muscle-derived cancers. Int J Mol Sci. (2023) 24:900. doi: 10.3390/ijms241511900, PMID: 37569275 PMC10418583

[26] Forni C Facchiano F Bartoli M Pieretti S Facchiano A D'Arcangelo D. Beneficial role of phytochemicals on oxidative stress and age-related diseases. Biomed Res Int. (2019) 2019:1–16. doi: 10.1155/2019/8748253, PMID: 31080832 PMC6475554

[27] Was H Dulak J Jozkowicz A. Heme oxygenase-1 in tumor biology and therapy. Curr Drug Targets. (2010) 11:1551–70. doi: 10.2174/1389450111009011551, PMID: 20704546

[28] Aguirre JD Culotta VC. Battles with iron: manganese in oxidative stress protection. J Biol Chem. (2012) 287:13541–8. doi: 10.1074/jbc.R111.312181, PMID: 22247543 PMC3340200

[29] Yamakura F Kawasaki H. Post-translational modifications of superoxide dismutase. Biochim Biophys Acta. (2010) 1804:318–25. doi: 10.1016/j.bbapap.2009.10.010 19837190

[30] von Ossowski I Hausner G Loewen PC. Molecular evolutionary analysis based on the amino acid sequence of catalase. J Mol Evol. (1993) 37:71–6. doi: 10.1007/BF00170464, PMID: 8360921

[31] Nandi A Yan LJ Jana CK Das N. Role of catalase in oxidative stress- and age-associated degenerative diseases. Oxidative Med Cell Longev. (2019) 2019:1–19. doi: 10.1155/2019/9613090, PMID: 31827713 PMC6885225

[32] Hasegawa Y Takano T Miyauchi A Matsuzuka F Yoshida H Kuma K. Decreased expression of glutathione peroxidase mRNA in thyroid anaplastic carcinoma. Cancer Lett. (2002) 182:69–74. doi: 10.1016/S0304-3835(02)00069-1, PMID: 12175525

[33] Ameziane El Hassani R Buffet C Leboulleux S Dupuy C. Oxidative stress in thyroid carcinomas: biological and clinical significance. Endocr Relat Cancer. (2019) 26:R131–43. doi: 10.1530/ERC-18-0476, PMID: 30615595

[34] Cazarin J Dupuy C Pires de Carvalho D. Redox homeostasis in thyroid Cancer: implications in Na. Int J Mol Sci. (2022) 23:129. doi: 10.3390/ijms23116129, PMID: 35682803 PMC9181215

[35] Ashtekar A Huk D Magner A La Perle KMD Boucai L Kirschner LS. Alterations in Sod2-induced oxidative stress affect endocrine Cancer progression. J Clin Endocrinol Metab. (2018) 103:4135–45. doi: 10.1210/jc.2018-01039, PMID: 30165401 PMC6194813

[36] Larkin J Ascierto PA Dréno B Atkinson V Liszkay G Maio M. Combined vemurafenib and cobimetinib in BRAF-mutated melanoma. N Engl J Med. (2014) 371:1867–76. doi: 10.1056/NEJMoa1408868, PMID: 25265494

[37] Sanz-Garcia E Argiles G Elez E Tabernero J. BRAF mutant colorectal cancer: prognosis, treatment, and new perspectives. Ann Oncol. (2017) 28:2648–57. doi: 10.1093/annonc/mdx401, PMID: 29045527

[38] Chen B Zhu Z Li L Ye W Zeng J Gao J. Effect of overexpression of Oct4 and Sox2 genes on the biological and oncological characteristics of gastric cancer cells. Onco Targets Ther. (2019) 12:4667–82. doi: 10.2147/OTT.S209734, PMID: 31417271 PMC6592062

[39] Fagman H Amendola E Parrillo L Zoppoli P Marotta P Scarfò M. Gene expression profiling at early organogenesis reveals both common and diverse mechanisms in foregut patterning. Dev Biol. (2011) 359:163–75. doi: 10.1016/j.ydbio.2011.08.015, PMID: 21924257 PMC3206993

[40] Lazzaro D Price M de Felice M Di Lauro R. The transcription factor TTF-1 is expressed at the onset of thyroid and lung morphogenesis and in restricted regions of the foetal brain. Development. (1991) 113:1093–104. doi: 10.1242/dev.113.4.1093, PMID: 1811929

[41] Mansouri A Chowdhury K Gruss P. Follicular cells of the thyroid gland require Pax8 gene function. Nat Genet. (1998) 19:87–90. doi: 10.1038/ng0598-87, PMID: 9590297

[42] Macchia PE Lapi P Krude H Pirro MT Missero C Chiovato L. PAX8 mutations associated with congenital hypothyroidism caused by thyroid dysgenesis. Nat Genet. (1998) 19:83–6. doi: 10.1038/ng0598-83, PMID: 9590296

[43] Chilczuk B Marciniak B Stochmal A Pecio Ł Kontek R Jackowska I. Anticancer potential and capsianosides identification in lipophilic fraction of sweet pepper. Molecules. (2020) 25:3097. doi: 10.3390/molecules25133097 32646039 PMC7412467

[44] Mori T Ohnishi M Komiyama M Tsutsui A Yabushita H Okada H. Growth inhibitory effect of paradicsompaprika in cancer cell lines. Oncol Rep. (2002) 9:807–10. doi: 10.3892/or.9.4.807 12066213

[45] Li S Cai Y Guan T Zhang Y Huang K Zhang Z. Quinic acid alleviates high-fat diet-induced neuroinflammation by inhibiting DR3/IKK/NF-κB signaling via gut microbial tryptophan metabolites. Gut Microbes. (2024) 16:2374608. doi: 10.1080/19490976.2024.2374608, PMID: 38972055 PMC11229714

[46] Aghababaei F Hadidi M. Recent advances in potential health benefits of quercetin. Pharmaceuticals. (2023) 16:1020. doi: 10.3390/ph16071020, PMID: 37513932 PMC10384403

